# Treatment of AO/OTA 43-C3 Pilon Fracture: Be Aware of Posterior Column Malreduction

**DOI:** 10.1155/2019/4265782

**Published:** 2019-04-14

**Authors:** Junjie Guan, Moran Huang, Qiuke Wang, Yunfeng Chen, Lei Wang

**Affiliations:** Department of Orthopaedics, Shanghai Jiaotong University Affiliated Sixth People's Hospital, 200233, China

## Abstract

Treatment of pilon fractures remains challenging due to the difficulty of fracture reduction and associated soft tissue complications. The aim of this study was to evaluate the pitfalls and strategies of posterior column reduction in the treatment of complex tibial pilon fractures (AO/OTA 43-C3). Thirteen AO/OTA classification 43-C3 type pilon fractures treated between January 2013 and January 2016 were retrospectively analyzed. Nine cases were treated by external fixation within 26 hours (range, 6–56 hours) after injury. The definitive open reduction and internal fixation (ORIF) was performed after the wound was healed without infection and soft tissue swelling had subsided. During the delayed/second-stage operation, the articular surface of the distal tibial plafond was reduced through the posterolateral and anterior approaches. X-ray and CT scans were performed pre- or postoperatively. The reduction quality was evaluated using Burwell–Charnley's radiographic criteria. The follow-up was performed routinely and all complications were recorded. Ankle function was evaluated using the American Orthopedic Foot and Ankle Society (AOFAS) ankle-hindfoot score. During the delayed/second-stage operation, primary reduction of the posterior column was performed entirely through posterolateral approaches. However, poor posterior column reduction was revealed by fluoroscopy in four cases, three of which were readjusted through the posterolateral and anterior approaches, and the fourth was adjusted directly through the anterior approach. Postoperative CT scan revealed that the step-off of the articular surface was less than 2 mm in 12 cases, and in only one case the step-off was greater than 2 mm but less than 5 mm. The satisfactory rate was 92.3% according to Burwell–Charnley's reduction criteria. Eleven patients were followed up regularly; superficial infections occurred in two cases but healed after wound care treatment in 3 and 5 weeks, respectively. All eleven fractures were healed within an average of 3.6 months (range, 2.6–5 months). The average range of ankle motion was 19° of dorsiflexion and 28° of plantar-flexion. The mean AOFAS ankle-hindfoot score was 82 (range, 61–92). In our opinion, we suggest that the reduction of the articular surface should be performed through combined posterolateral and anterior approaches in a delayed operation, with flexible fixation of the posterior column. If the posterior column is poorly reduced, the articular surface can easily be manipulated through anterior approaches. According to this strategy, satisfactory outcomes of AO/OTA C3 pilon fractures would be anticipated.

## 1. Introduction

Pilon fractures often involve an axial load mechanism that leads to joint surface destruction and remain challenging for most orthopedic surgeons. Pilon fractures have specific characteristics including massive soft tissue swelling, joint surface destruction, and open wounds. Rüedi and Allgöwer originally outlined the treatment principles of pilon fractures, which are open reduction and rigid internal fixation with plates and screws [1]. However, subsequent studies revealed a number of complications, including wound dehiscence, superficial and deep infections, osteomyelitis, and nonunion, following such treatment for pilon fracture [2, 3]. These complications have led many surgeons to choose external fixation as primary stage surgery. After the soft tissue swelling subsidies, definitive internal fixation can then be performed [4]. 

Although the risk of soft tissue complications decreases with the two-stage surgery protocol, reduction of the joint surface remains a problem. To better restore the articular surface, different fixation methods have been advocated. There is still some confusion in the literature as to the best treatment strategy for AO classification-type C3 pilon fractures [4–6]. Ketz reported that the addition of a posterolateral approach ensured accurate reduction of the tibiotalar joint [5]. Chan et al. reported that there was no statistically proven benefit to combined surgical approaches to treat tibial pilon fractures with regard to the quality of articular reduction. However, the addition of the staged posterior approach significantly increased the risk of nonunion [4].

Anatomical articular reduction should be achieved at the time of definitive open reduction and internal fixation (ORIF). The best way to handle the articular fragments in complex pilon fractures remains challenging. We found that lack of anatomic reduction of the posterior column would impede the desired reduction of the articular surface of the distal tibial plafond. Although reduction of the posterior column can be performed through a posterolateral approach, there are still some pitfalls with regard to the congruency restoration of the articular surface. In this article, we propose that reduction of the posterior column should be performed through combined posterolateral and anterior approaches during a delayed/second-stage operation.

## 2. Materials and Methods

The ethics committee of Shanghai Jiao Tong University Affiliated Sixth People's Hospital approved the present study. Thirteen pilon (AO/OTA 43-C3) fractures treated between January 2013 and January 2016 were retrospectively reviewed. The patients were six men and seven women, ranging in age from 32 to 64 years (average, 46 years). The mechanisms of injury were falls from heights (five) and motor vehicle accidents (eight). Of the thirteen patients, five (33.3%) had associated injuries, including two cases of craniocerebral trauma and three rib fractures. Three patients had open fractures, which were debrided within eight hours after injury. Four patients were treated with calcaneal bone traction in the emergency room. The other nine patients were treated by external fixation including the two open fracture cases during debridement.

After initial physical examination in the emergency department, the limb was temporarily stabilized with a well-padded splint. Initial external fixation was performed 26 h (range, 6–56 hours) after injury. During the operation, a thigh pneumatic tourniquet was applied to reduce bleeding only in the debridement. The proximal and distal pins were inserted well away from the subsequent surgical incision. The proximal pins were inserted into the tibia, and the two distal pins were inserted into the metatarsals and calcaneus. The length, alignment, and rotation of the distal tibia were restored preliminarily by maneuvers. The external fixator might be fastened to maintain the reduction in the first-stage. The injury characteristics and first-stage treatment are detailed in Table 1.

**Figure 1:**
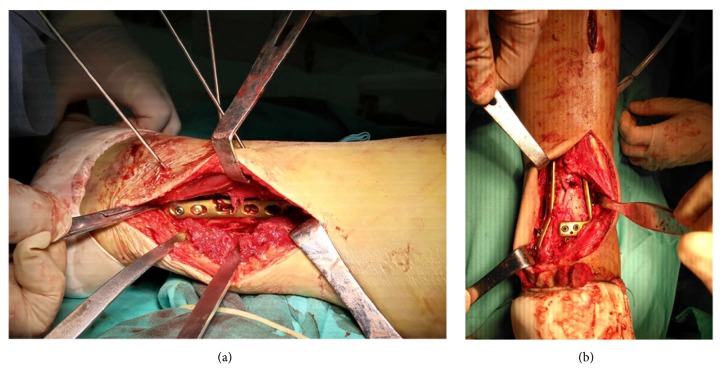
(a) A posterolateral and an anterior extensile approach were adopted for the posterior column and anterior column reduction, respectively.

The timing of the delayed/second-stage operation was dependent on soft tissue healing. When the swelling around the ankle joint subsided and the skin wrinkle sign was observed, as well as three consecutive normal results of routine blood tests and CRPs, the delayed/second-stage operation could be performed. The senior attending doctor (Lei Wang) performed the surgery. The patient was placed in a “floating position” with all bony prominences padded [7]. A thigh pneumatic tourniquet was applied to reduce bleeding. The limb was sterilized from the level of the tourniquet to the toes. A posterolateral incision was created over the ankle joint (Figure 1(a)), exposing both the fractures of the fibula and the posterior column. Then the soft tissue was released and the incarcerated bony callus was removed. The fibula was reduced anatomically and fixed with a plate under direct visualization. The posterior column was exposed via the same incision. Through the posterolateral approach, the cortex alignment of the posterior column was reconstructed first, as a reference for the reduction. However, the congruency of the articulation was difficult to judge by the C-arm. After the posterior column was temporarily fixed with a plate, the patient was turned into the supine position. An anterior extensile approach of the ankle joint was adopted, and the frontal articular surface was exposed (Figure 1(b)). The impacted “die-punch” fragments should be reduced first, then the medial malleolus and the “chaput” fragment were reduced sequentially. Autogenous bone graft or bone graft substitute material was used to support the reduction of the osteochondral fragments. The reduction of the articular surface and the length and axial alignment of the lower limbs were checked by C-arm. After that, the rafting plate was applied anteriorly, and an additional miniplate could be used when necessary. As soon as the reduction and fixation were found to be satisfactory by fluoroscopy, both the posterolateral and anterior incisions were closed after adequate saline irrigation.

Postoperatively, a posterior short leg splint with the ankle joint in neutral position was applied immediately to release the incision tension; the splint could be removed 3 weeks later. Then active motion exercises were encouraged and toe-touch weight bearing was allowed. Partial weight bearing was allowed when early callus formation was observed radiographically, usually at 6 weeks postoperatively. CT scan and X-ray were used routinely to examine the reduction quality after the operation. During the follow-up, X-rays were also taken at standard time intervals of 2 weeks, 6 weeks, 3 months, 6 months, and 12 months. The radiological imaging was reviewed by the senior author according to the reduction criteria of Burwell–Charnley [8]. All complications (infections, nonunion, delayed union, malunion, and failure of fixation) were carefully observed and the active range of ankle motion was measured with a goniometer at each follow-up visit. The American Orthopedic Foot and Ankle Society (AOFAS) score was used to assess ankle function.

## 3. Results

The mean interval between injury and first-stage operation was 26 hours (range, 6–56 hours). The interval between injury and delayed/second-stage surgical fixations averaged 18 days (range, 7–24 days). The mean duration of the delayed/second-stage operation was 126 minutes (range, 96–168 minutes).

**Figure 2:**
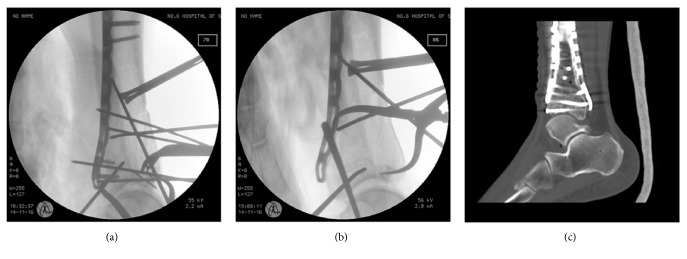
(a) The posterior column moved forward and the reduction was unsatisfactory. (b) After reduction of the anterior column, the posterior column was restored and the congruency of the ankle joint was satisfactory. (c) CT showed that the articulation and congruency were restored.

During the operation, we encountered four cases of malreduction on the posterior column, which were identified by fluoroscopy only after the entire reduction of the articular surface through the anterior approach (Figure 2(a)). All these four cases were characterized by severely impacted “die-punch” fragments. In three of these cases fixed posteriorly with a 3.5 mm locking compression plate (LCP), we went back to the posterolateral approach, took off the locking screws fixed in the distal part of the posterior column, then readjusted the articular surface through the anterior approach (Figure 2(b)). In the remaining case with a miniplate fixed posteriorly, we accomplished readjustment only through the anterior approach without any intervention through the posterior approach. Postoperative CT examination showed that the reduction of the articular surface was perfect with step-off less than 2 mm in 12 cases (Figure 2(c)), except for one case in which readjustment was performed only anteriorly, in which the step-off was greater than 2 mm but less than 5 mm. According to the scale described by Burwell and Charnley, 12 fractures had anatomic reduction (92.3%) and one was fair; no poor reduction was observed.

**Figure 3:**
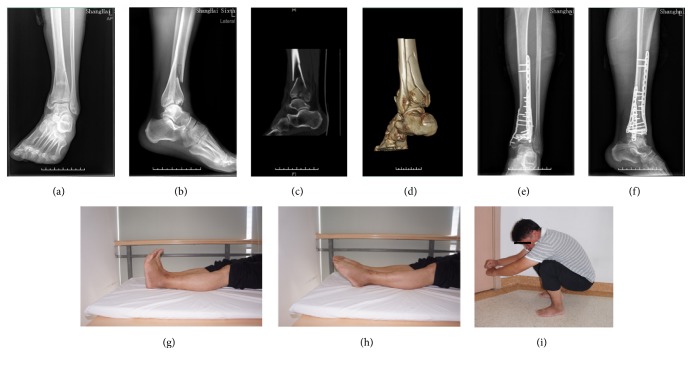
The image and functional outcome of a typical patient. Preoperative imaging: (a, b) X-ray; (c, d) CT. Postoperative imaging: (e, f) X-ray. The range of ankle movement: (g) dorsiflexion; (h) plantarflexion; (i) squat.

Eleven patients were followed up regularly, but two patients were lost to follow-up. The mean clinical follow-up duration was 32 months (range, 24–45 months). There were no cases of deep infection, loss of reduction, or fixation failure during the follow-up period. Two superficial infections after partial necrosis of the incision were successfully treated with antibiotics and wound care treatment. The images of a typical patient are shown in Figure 3: X-ray (Figures 3(a) and 3(b)) and CT (Figures 3(c) and 3(d)). The mean bone union time was 3.6 months (range, 2.6–5 months) (Figures 3(e) and 3(f)). The range of ankle dorsiflexion and plantarflexion was 19° (range, 5–23°) and 28° (range, 16–34°) (Figures 3(g) and 3(h)), respectively. The patient was able to squat fully after the operation (Figure 3(i)). The average AOFAS score was 82 (range, 61–92). The main outcome measures are shown in Table 2.

## 4. Discussion

Treatment of high-energy pilon fractures is challenging because of the difficulty of anatomic reduction and the risk of associated complications [9, 10]. Currently, no standard treatment protocol is available for high-energy pilon fractures especially for those classified as AO/OTA 43-C3, in which the articular surface is fractured into multiple fragments and impacted severely. Surgical treatment is important in the management of pilon fracture and orthopedic surgeons have strived to improve the outcome. However soft tissue complications can lead to poor results such as infection and wound dehiscence regardless of the high quality of the anatomic reduction. A staged protocol for the treatment of severe, high-energy pilon fractures has been reported [11, 12]. In the initial stage, external fixation is performed to alleviate soft tissue swelling. In the delayed/second stage, patients are treated with ORIF to achieve anatomic reduction of the articular surface and the correct alignment of the metaphysis. Following application of these protocols, soft tissue complications decreased significantly. However, the recovery of pilon fracture is still unsatisfactory, and the best way to deal with the comminuted articular surface of the distal tibial plafond is still controversial.

The management of pilon fractures has changed with the overall understanding of such fractures. The current treatment strategy takes into consideration not only the condition of the soft tissues, but also the reduction quality and fracture healing. Some surgeons choose fibular and posterior column internal fixation at the time of first-stage surgery. However, for articular comminuted C3 fractures, limited internal fixation in the initial stage may impede later anatomic reduction of the ankle joint [5]. Therefore, many surgeons are inclined to choose external fixation initially, simply to maintain the length and axial alignment of the lower extremity [13, 14]. Once the condition of the soft tissue allows, the definite surgery can be performed. To obtain articular surface anatomical reduction, a posterolateral approach followed by a direct anterior approach was reported in an early clinical study [5]. After a minimum 1-year follow-up, the articular reduction and ankle function score improved in the staged posterior tibial plating group much better than in the direct anterior tibial plating group. However, after prolonged clinical study, Chan et al. reported that addition of the staged posterior approach did not benefit the articular reduction but significantly increased the risk of nonunion [4]. Consequently, the best way to balance the articular reduction with the multiple complications of fracture healing and ankle function should be further explored.

In this article, we propose that AO/OTA 43-C3 pilon fractures should first be treated with external fixation or calcaneal traction. Only after the soft tissue condition allows should delayed ORIF be performed definitively. In the delayed/second-stage operation, the reduction of the articular surface is achieved through the posterolateral combined with the anterior approach. We reduced and fixed the fibular fracture using the posterolateral incision in the first instance. Using the same incision, the posterior column was then reduced and fixed by temporary plating. Then the anterior extensile approach of the ankle joint was adopted. The posterolateral and anterior incisions were approximately 7 cm apart. Reduction of the impacted “die-punch” fragments and the anterior column was performed through the anterior approach. However, poor posterior column reduction with articular surface step-off was unexpectedly discovered in four patients during the operation, accounting for 30.8% of all patients. This might be the reason that, in cases where the “die-punch” fragments impact severely in AO/OTA type C3 fractures, the posterior column fragments might be pushed forward or angulated sagittally after posterior plating. Consequently, in one of these four cases, the poor posterior column reduction was adjusted immediately through the anterior approach, which resulted in a residual step-off postoperatively on the articular surface of more than 2 mm but less than 5 mm. We speculated that this may be due to the fibular plate obscuring the image display on the lateral view of the articular surface by intraoperative fluoroscopy. As for the three others patients with poor posterior column reduction, we returned to the posterior column through the posterolateral approach, removed partial screws on the distal posterior fragments, and then readjusted the entire articular surface through both the posterolateral approach and the anterior approach in the same procedure. According to the postoperative X-ray and CT scans, the articular surface step-off was less than 2 mm in 12 patients including these three patients, and the anatomic reduction rate was achieved in 92.3% according to Burwell and Charnley's criteria.

Because the articular surface could be reduced definitely only by direct exposure through the anterior approach, the first manipulation on the posterior column through the posterolateral approach might result in malreduction on the posterior part of the articular surface. High-quality articular reduction reduces the rate of arthritis and joint fusion [15]. Combined approaches provide sufficient articular visualization, facilitating both reduction and fixation. However, one study reported that the combined approaches possibly increased the risk of fracture healing and soft tissue complications [4]. The reason may be that the combined approach increases surgical stripping and damage to the blood supply of bone tissue. The mean union time in our series was 3.6 months, which was comparable to that of a previously published study [16]. We suggest that the soft tissue and periosteum detachment should be minimal. Two patients developed a superficial infection. No cases of deep infections, wound necrosis, or skin sloughing were observed during the follow-up period. The complication rate was comparable with the data in the literature [17], and the AOFAS score was also satisfactory.

Generally, the single anterior approach may provide adequate exposure of the anterior column but causes much difficulty in manipulating the posterior column fragment to achieve anatomic reduction anteriorly in the delayed operation, which might result in the step-off or separated displacement of the articular surface. A ministep-off or separated displacement would be missed by intraoperative fluoroscopy. However, as a consequence the outcome of a pilon fracture significantly worsened and the incidence of posttraumatic osteoarthrosis obviously increased. Alternatively, the combined approaches provide direct exposure of both the posterior column fragment and articular surface. The best and most accurate reduction of the articular surface may be accomplished through both approaches in the same procedure. When combined with soft tissue protection measures, including minimum stripping of fragments, we suggest that the combined approaches should be employed for delayed open reduction and internal fixation of AO/OTA C3 pilon fractures. Meanwhile, flexible fixation of the posterior column would benefit the final reduction of the articular surface; thus a satisfactory outcome will be anticipated.

There are several limitations to our study. First, there was no comparison with any other treatment option. Second, it was a retrospective study and selection and assessor biases are always possible. Finally, the number of patients in our study was relatively small and the follow-up period was short. These issues will be addressed in future studies.

## 5. Conclusions

In our opinion, if there is no anatomic cortical reference for posterior column reduction, we suggest flexible fixation on posterior plating, with either a miniplate or even no screw fixation of the distal fragment, which would benefit the subsequent reduction of the “die-punch” fragments and the anterior column through the anterior approach. In addition, the combination of the posterolateral and anterior approaches in the same procedure provides an opportunity to readjust a malpositioned posterior column, which is useful to achieve anatomic reduction of the articular surface in AO/OTA 43-C3 pilon fracture treatment.

## Figures and Tables

**Table 1:** Injury characteristics and first-stage treatment.

N.	Sex/Age	Injury mechanism	Open/Close	External fixation/Traction	Waiting days
1	F/32	Fall	Close	Traction	7
2	F/38	Motor vehicle	Open	External fixation	22
3	M/36	Fall	Close	Traction	12
4	F/43	Fall	Close	External fixation	21
5	F/41	Motor vehicle	Open	External fixation	24
6	M/46	Motor vehicle	Close	Traction	15
7	F/44	Fall	Close	External fixation	20
8	M/49	Motor vehicle	Close	External fixation	21
9	F/48	Fall	Close	External fixation	19
10	M/52	Motor vehicle	Open	Traction	16
11	F/51	Motor vehicle	Close	External fixation	16
12	M/64	Motor vehicle	Close	External fixation	22
13	M/53	Motor vehicle	Close	External fixation	19

**Table 2:** Clinical and functional outcomes.

No	Operation Time (mins)	Malreduction on Posterior Column	Articular Step-off	Fracture Healing mons	Complications	AOFAS	Follow up (mons)
1	96	N	<2mm	2.8	/	89	28
2	102	N	<2mm	3	/	92	30
3	112	N	<2mm	3.2	/	81	24
4	124	Y	<2mm	3.4	/	83	30
5	130	N	<2mm	3.1	/	85	32
6	138	N	<2mm	3.2	superficial infections	66	34
7	106	N	<2mm	4.1	/	80	32
8	142	Y	<2mm	4.0	/	90	34
9	136	N	<2mm	4.2	superficial infections	91	30
10	168	Y	2-5mm	4.2	/	61	45
11	140	N	<2mm	5	/	81	32
12	138	Y	<2mm	-	/	-	-
13	112	N	<2mm	-	/	-	-

## Data Availability

The data used to support the findings of this study are included within the article.
